# What global satisfaction scores miss: decision-making remains the weakest domain in intensive care unit care

**DOI:** 10.62675/2965-2774.20260075

**Published:** 2026-07-24

**Authors:** Gabriela André de Souza, Luccas Ribeiro Mesquita, Renata Rego Lins Fumis, Eduardo Lyra de Queiroz, Laerte Pastore, Eduardo Leite Vieira Costa, Fernando José da Silva Ramos

**Affiliations:** 1 Hospital Sírio-Libanês Intensive Care Department São Paulo SP Brazil Intensive Care Department, Hospital Sírio-Libanês - São Paulo (SP), Brazil.; 2 Universidade Federal de São Paulo Escola Paulista de Medicina Department of Anesthesiology, Pain and Intensive Care São Paulo SP Brazil Department of Anesthesiology, Pain and Intensive Care, Escola Paulista de Medicina, Universidade Federal de São Paulo - São Paulo (SP), Brazil.

## INTRODUCTION

Quality assessment in intensive care units (ICUs) cannot rely solely on clinical indicators and survival outcomes. The experience of patients and their families is a central component of humanized care, and family satisfaction has become a relevant quality indicator, as it reflects communication, emotional support, and engagement during periods of high vulnerability.^(1)^

To be useful as a comparable and actionable metric, family satisfaction must be assessed using standardized instruments. The Family Satisfaction in the Intensive Care Unit questionnaire (FS-ICU 24R) is one of the most widely used tools to measure family perceptions of care and participation in decision-making.^(2,3)^

The Net Promoter Score (NPS) is a widely used single-item measure of willingness to recommend healthcare services.^(4)^ Recently, the NPS has been used to evaluate family satisfaction in the pediatric ICU.^(5)^ However, global satisfaction scores may mask important domain-specific gaps, particularly in communication and shared decision-making.^(5)^

In this letter, we report findings from a longitudinal study evaluating family satisfaction after ICU discharge using the FS-ICU 24R and its correlation with the NPS. We hypothesized that, if strongly correlated with FS-ICU scores, the NPS could serve as a rapid screening tool for monitoring family satisfaction.

However, global satisfaction measures may fail to capture domain-specific aspects of family experience, particularly those related to communication and decision-making. Understanding whether simplified metrics, such as NPS, adequately reflect these dimensions remains an important question.

## METHODS

We evaluated 140 family members of patients who had an ICU stay of at least 48 hours at a tertiary private hospital in São Paulo, Brazil. We used the culturally adapted FS-ICU 24R^(6)^ and NPS from December 2024 to August 2025. The FS-ICU 24R instrument generates scores for overall satisfaction, satisfaction with care, and satisfaction with decision-making. The score was rescaled to 0 - 100, with higher scores indicating greater satisfaction. The NPS was collected simultaneously to capture global willingness to recommend the ICU. Descriptive statistics, analysis of variance, and Pearson's and Spearman's correlation analyses were performed. Data were analyzed using JASP (version 0.95.04, 2025), and p values < 0.05 were considered statistically significant.

Ethics approval was obtained from the Research Ethics Committee of *Hospital Sírio-Libanês*, and all participants provided written Informed Consent.

## RESULTS

During the study period, the ICU recorded 863 admissions. Patients had a median age of 74 years, 42.9% were female, the median ICU length of stay was 3.8 days, and the mean Simplified Acute Physiology Score 3 was 49.8 ± 14.6. Intensive care unit mortality was 3.7% (32/863).

A total of 140 (16.2%) family members of patients with an ICU stay ≥ 48 hours were evaluated. Among family respondents, 98/140 (70.0%) were female, with a mean age of 59.1 ± 14.6 years. Previous ICU hospitalization of a family member was reported by 74.3% of respondents. Regarding the relationship to the patient, 46.5% were spouses and 37.1% were the patients’ children. More than half of respondents (60.7%) were living with the patient at the time of ICU admission.

Overall satisfaction was high (mean 83.4 ± 15.2), and satisfaction with care by FS-ICU 24R was also high (mean 92.1 ± 18.6). In contrast, satisfaction with decision-making by FS-ICU 24R consistently scored lower (mean 74.6 ± 15.2), indicating that even families who were globally satisfied with the ICU experience had concerns about participation, clarity, and support during clinical decision-making. The NPS reached 86.4, with 89.3% of respondents classified as promoters.

**Figure 1:**
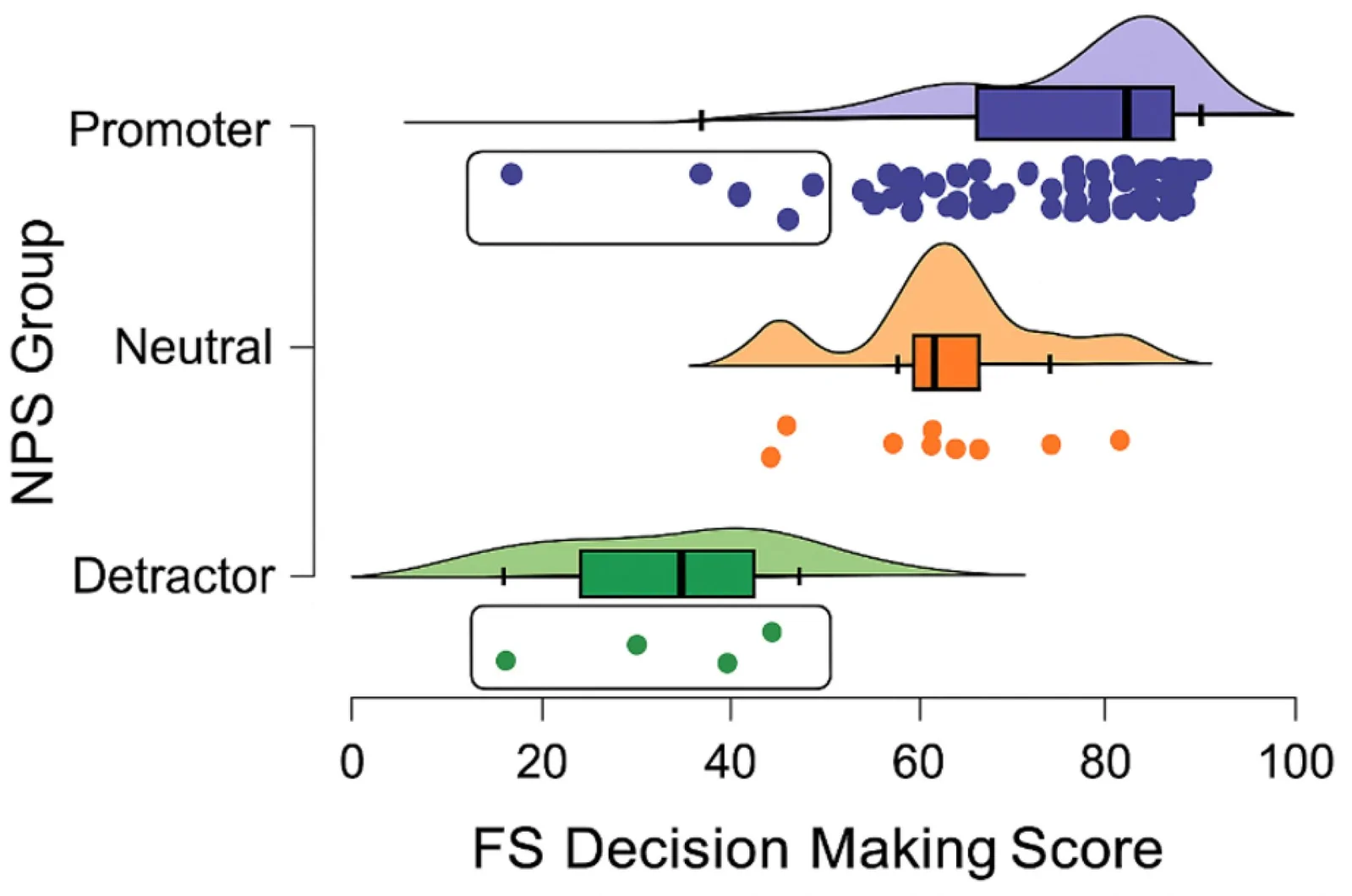
Distribution of Family Satisfaction in the Intensive Care Unit 24R decision-making scores across Net Promoter Score groups. Notably, lower satisfaction with decision-making persists even among families classified as Net Promoter Score promoters (dots inside the rectangles).

The NPS showed a moderate to strong correlation with the FS-ICU 24R overall score (Pearson's r = 0.637; Spearman's ρ = 0.546; both p < 0.001). However, domain-level analysis revealed important discrepancies: even among families classified as NPS promoters, lower decision-making scores persisted (Figure 1).

NPS - Net Promoter Score; FS - Family Satisfaction in the Intensive Care Unit 24R.

## DISCUSSION

These findings highlight a limitation of global satisfaction metrics, which can provide a misleading sense of quality. While the NPS provides a rapid overview of overall experience, it may fail to capture key aspects of family involvement in decision-making, in which lower satisfaction persists despite high global ratings. Decision-making is consistently reported as a challenging domain in ICU family experience.^(7)^

From a quality-improvement perspective, global metrics such as the NPS should not be used in isolation, as they may mask actionable gaps in care that are captured only by domain-specific instruments.

In this single-center study, high global satisfaction did not ensure uniformly positive experiences across domains. The FS-ICU 24R identified lower satisfaction with decision-making, even among NPS promoters, reinforcing the need for domain-specific tools to guide targeted improvements.

## Data Availability

After publication the data will be available on demand to authors.
